# Comparative Transcriptome Analysis Unveils the Molecular Mechanism Underlying Sepal Colour Changes under Acidic pH Substratum in *Hydrangea macrophylla*

**DOI:** 10.3390/ijms232315428

**Published:** 2022-12-06

**Authors:** Razieh Rahmati, Rasmieh Hamid, Zahra Ghorbanzadeh, Feba Jacob, Pezhman Azadi, Mehrshad Zeinalabedini, Laleh Karimi Farsad, Mehrbano Kazemi, Mohammad Ali Ebrahimi, Fahimeh Shahinnia, Ghasem Hosseini Salekdeh, Mohammad Reza Ghaffari, Mohammad Reza Hajirezaei

**Affiliations:** 1 Department of Systems Biology, Agricultural Biotechnology Research Institute of Iran (ABRII), Agricultural Research, Education and Extension Organization (AREEO), Karaj 3135933151, Iran; 2 Department of Plant Breeding, Cotton Research Institute of Iran (CRII), Agricultural Research, Education and Extension Organization (AREEO), Gorgan 4969186951, Iran; 3 Centre for Plant Biotechnology and Molecular Biology, Kerala Agricultural University, Thrissur 680656, India; 4 Department of Genetic Engineering, Agricultural Biotechnology Research Institute of Iran (ABRII), Agricultural Research, Education and Extension Organization (AREEO), Karaj 3135933151, Iran; 5 Department of Biotechnology, Payame Noor University, Tehran 1599959515, Iran; 6 Bavarian State Research Center for Agriculture, Institute for Crop Science and Plant Breeding, 85354 Freising, Germany; 7 Department of Molecular Sciences, Macquarie University, North Ryde, NSW 2109, Australia; 8 Department of Physiology and Cell Biology, Leibniz Institute of Plant Genetics and Crop Plant Research, 06466 Gatersleben, Germany

**Keywords:** hydrangea, acidic pH, transcriptome, anthocyanin, flavonoid, blue colour, *HymMYB2*

## Abstract

The hydrangea (*Hydrangea macrophylla* (Thunb). Ser.), an ornamental plant, has good marketing potential and is known for its capacity to change the colour of its inflorescence depending on the pH of the cultivation media. The molecular mechanisms causing these changes are still uncertain. In the present study, transcriptome and targeted metabolic profiling were used to identify molecular changes in the RNAome of hydrangea plants cultured at two different pH levels. De novo assembly yielded 186,477 unigenes. Transcriptomic datasets provided a comprehensive and systemic overview of the dynamic networks of the gene expression underlying flower colour formation in hydrangeas. Weighted analyses of gene co-expression network identified candidate genes and hub genes from the modules linked closely to the hyper accumulation of Al^3+^ during different stages of flower development. *F3′5′H*, *ANS*, *FLS*, *CHS*, *UA3GT*, *CHI*, *DFR*, and *F3H* were enhanced significantly in the modules. In addition, *MYB*, *bHLH*, *PAL6*, *PAL9*, and *WD40* were identified as hub genes. Thus, a hypothesis elucidating the colour change in the flowers of Al^3+^-treated plants was established. This study identified many potential key regulators of flower pigmentation, providing novel insights into the molecular networks in hydrangea flowers.

## 1. Introduction

Flower colour, one of nature’s most magnificent displays, plays an important role in attracting animal pollinators, and is therefore crucial for plant ecology and evolution [1]. Among the various plant pigments, carotenoids and flavonoids are the most common and diverse types [2]. However, apart from attracting pollinators, anthocyanins and other flavonoid compounds that share a common biosynthetic pathway may also act as defensive agents or compounds that protect against various biotic and abiotic stresses [3]. Thus, pathogens, seed predators, and herbivores could act as non-pollinator agents for flower colour selection [4]. Variation in flower colour may be influenced by abiotic factors such as soil chemistry and heat and drought stresses [5]. The colour of hydrangeas is highly dependent on the pH of the soil in which they grow [6]. The acidic pH of the soil or exogenous addition of Al^3+^ can cause colour alteration in some infertile flowers of Hydrangea [7]. The genus Hydrangea of the hydrangea family, native to Japan, has been divided into two sections: *Hydrangea* McClint. and *Cornidia* Engl, which harbour a total of 25 species and numerous subspecies [8]. Furthermore, numerous cultivars have been developed and labelled as species, complicating this family’s taxonomy [9]. The majority of the species in the genus are shrubs; however, there are some arboreal forms, such as, *H. japonica*, and evergreen climbers, such as *H. petiolaris*. Because of their characteristic umbellate inflorescences, many of these species are sold as cut flowers [10]. According to the University of Tennessee, hydrangea was the second most widespread deciduous shrub in all horticulture markets in the United States in 2014 [11], with sales of more than 10 million plants (USD 91.2 million). Various hydrangea cultivars are derived from *Hydrangea macrophylla* (Thunb.) Ser. [12]. The capability of these species to change sepal colour in response to the pH of the growing medium is widely known in floriculture, but the underlying molecular mechanisms are still unknown [13]. According to Kikelly (2006), the development of blue colour is the highest in acidic soils, and the degree of blue colouration depends on the amount of aluminum available and the ability of a particular cultivar to absorb it [14]. Robinson et al. (1932) reported that both Al and anthocyanin (delphinidin-3-monoglucoside) are involved in the blue colouration of hydrangea sepals [15]. Takeda et al. (1985) showed that the blue colouration of hydrangea sepals was mainly due to the delphinidin-3-glucoside-aluminum-3-caffeoylquinic acid complex, with Al serving as a stabilizer [16]. Most pigments, chiefly secondary metabolites of the flavonoid pathway, accumulate in the vacuoles of flowering plants. Anthocyanin, the most common floral pigment, is a large polar molecule that is transported into the vacuole to accomplish its biological function [17,18]. The biosynthesis of approximately 20 different basic anthocyanins is mediated by biosynthetic and regulatory genes [18]. The anthocyanin delphinidin-3-glucoside, along with various co-pigments such as 5-O-p-coumaroylquinic acid, neochlorogenic acid, chlorogenic acid, and Al^3+^, contributes to the exquisite range of colours of hydrangea sepals, which ranges from blue to purple to red. When the composition of protoplast extracts from blue and red sepals was evaluated, it was found that the molar ratio of neochlorogenic acid, 5-O-p-coumaroylquinic acid, and Al^3+^ to delphinidin 3-glucoside was higher in the blue cells than in the red ones [19,20]. 

Aluminum (Al) is soluble in a toxic form, Al^3+^, in acidic soils (below pH 5.0) and is harmful to the cultivation of many crops, with root growth inhibition being the major symptom. Tolerance to Al has evolved in plants by a wide range of mechanisms that include the production of organic anions from root tips or through Al protein transporters [21]. Many Al transporters have been recognized in hydrangea shrubs, such as *HmVALT* and *HmPALT1*, which belong to the aquaporin superfamily (although *HmPALT1* is found only in sepals), and *HmPALT2*, an anion permease found in all hydrangea tissue [11,22]. Blom et al. (1992) reported that there was a positive correlation (r = 0.74) between the blue stain value and the Al leaf concentration of the two uppermost expanded leaves of *Hydrangea macrophylla* [23]. Transcriptome sequencing is now extensively utilized in plant research due to its speed, low price, and efficiency [24,25]. Xue et al. created the transcriptome profile of *Lonicera japonica* flowers at six developmental stages and constructed the regulatory networks of flower pigmentation pathways using weighted gene co-expression network analysis to examine the molecular and metabolic basis of pigmentation at various developmental stages. Transcriptomics data revealed that anthocyanins and chlorophylls were responsible for the early flower hues, while carotenoids were responsible for late golden flower colours [26]. The mechanisms regulating yellow colouration in tree peony (*Paeonia suffruticosa* Andr.) flowers were revealed by combining full-length transcriptomics and metabolomics [27]. In another study, transcriptomic and metabolomic analyses revealed that mutations in the coding regions of *ScCHI1/2* and *ScbHLH17* prevented the formation of anthocyanin in yellow and white *Senecio cruentus* cultivars. Differences in the branched metabolic flux of pelargonidin (Pg), cyanidin (Cy), and delphinidin (Dp)-type pathways are determined by the competition for naringenin between *ScF3′5′H*, *ScDFR1/2*, and *ScF3′H1* [28]. After studying the transcriptome, Chen, et al. [29] observed that Al exposure upregulated 730 genes in the leaves and 4287 genes in the roots of hydrangea, while it downregulated 719 genes in the leaves and 236 genes in the roots. From the data of metabolomic and transcriptomic analyses of *S. miltiorrhiza* flowers, a total of 100 unigenes that coded for 10 enzymes were recognized as the candidate genes linked with anthocyanin production. Decreased ANS gene expression lowered the anthocyanin content but led to an increased buildup of flavonoids in *S. miltiorrhiza* flowers [30]. Other studies have reported a connection between colour expression and DNA methylation in other species. A recent study using molecular markers of SSR and MSAP suggests that DNA methylation may be part of the molecular mechanism causing the change in the colour of hydrangea sepals in response to acidic pH [11]. Hyper methylation of the *MdMYB10* promoter initiates striped colouration due to an increased anthocyanin concentration in *Malus domestica* fruit. Alternatively, varying amounts of promoter methylation of the anthocyanindin synthase gene resulted in varied red or white flower colouration in the ornamental plant *Nelumbo nucifera* [31,32].

Since transcriptome and targeted metabolomic technologies have proven to be powerful tools for elucidating the mechanisms of colouration in various ornamental plants, we used these technologies to investigate differentially expressed genes (DEGs) during different developmental stages in the infertile flowers of Al^3+^-treated hydrangea and thus to elucidate the molecular pathways driving colour alteration. Global gene expression profiles were examined, with an emphasis on genes involved in anthocyanin biosynthesis and flavonoid metabolism, and the regulatory networks were established. This is the first comprehensive transcriptome and metabolome study of hydrangea flower colour variation at acidic pH. These discoveries will aid in the breeding of multi-coloured hydrangea and several other hydrangea species, as well as in the functional characterisation of genes and proteins of interest. 

## 2. Results

### 2.1. Change of Sepal Colour under Different Soil pH

**Figure 1:**
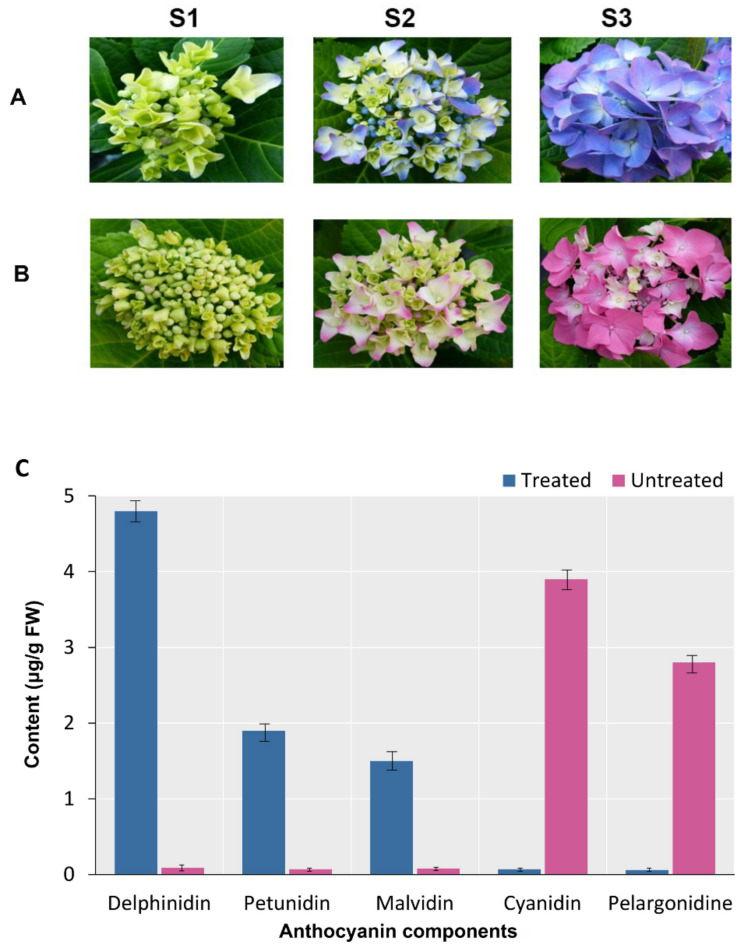
Different development stages of *Hydrangea macrophylla*. (**A**): The sepal of plants grown in acidic soil changed from pale yellow to blue–violet. (**B**): The sepal of plants grown in untreated soil changed from pale yellow to pink. (**C**): Anthocyanins contents of sepal in S3. Bars are the mean of triplicate determinations and standard error.

The sepal of plants grown in acidic soil (enriched with aluminum sulfate) changed from pale yellow at stage (I) or early flowering (EB, S1) to blue–violet at stage (S3) or full flowering (FB), as shown in Figure 1A. In the plants grown in untreated soil (control group, C), sepals were initially pale yellow at stage (I) or early flowering (EB). At stage (II, S2) or mid-flowering (MB), the margins of the sepals turned light pink, and at full flowering, pink (Figure 1B). In addition, several common types of anthocyanidins comprising cyanidin, delphinidin, malvidin, pelargonidin, and petunidin, involved in colour development, were quantified. High levels of delphinidin, petunidin, and malvidin were found in TS3, with levels of 4.8 µg/g fresh weights (FW), 1.9 µg/g FW, and 1.5 µg/g FW, respectively. In contrast, high levels of cyanidin and pelargonidin were detected in the pink flowers of CS3 (Figure 1C). This suggests that treatment causes differential accumulation of anthocyanidin content.

### 2.2. Transcriptome Sequencing, Annotation, and Analysis of DEGs

Using RNA-Seq, a sum of 72 million reads with a total nucleotide count of 328,854,166 bp (38.24 GB) was obtained. A total of 34.79 Gb of clean reads was acquired after cleaning and quality verification, with each library producing at least 5.78 Gb of clean reads. Q30 percentages were 91.76%, 92.89%, 90.94%, 91.87%, 93.91%, and 90.98%, respectively. These findings demonstrated that the quality of RNA-Seq was suitable for further investigation (Table S2). Successively, the de novo assembly produced 342,068 contigs and 186,477 unigenes, with an N50 of 903 nt and 794 nt. There were 109,316 unigenes between 200 and 500 nt (58.61%), 48,027 unigenes between 500 and 1000 nt (25.75%), and 7,459 unigenes longer than 2000 nt (4%) (Table 1).

### 2.3. Functional Annotation

**Figure 2:**
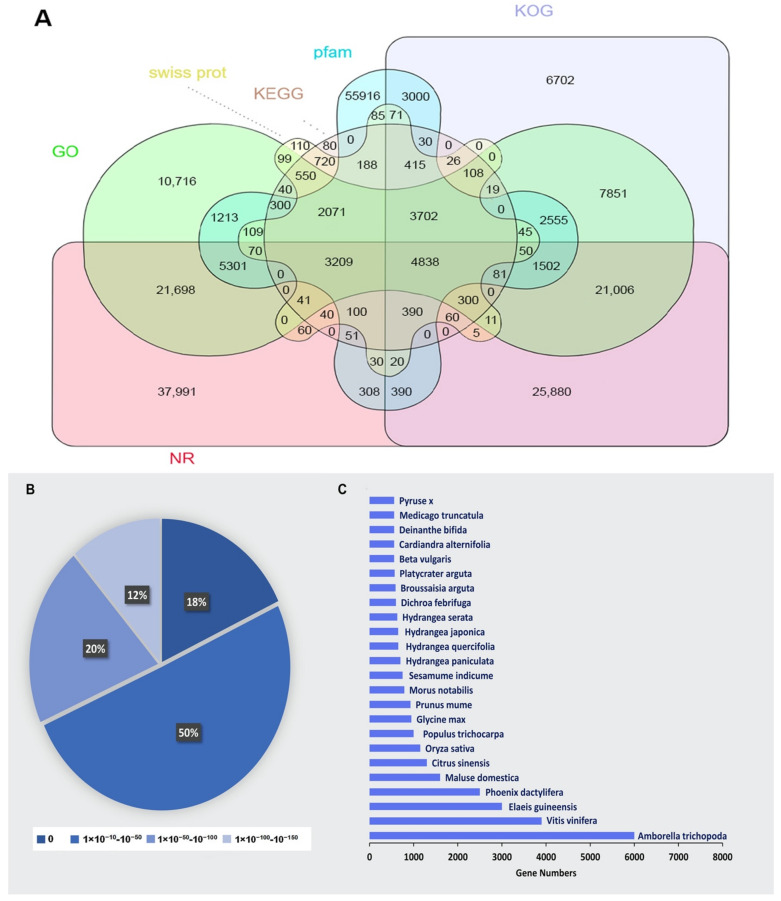
Characteristics of homology search of *Hydrangea macrophylla* unigenes. (**A**): Venn diagram of the number of unigenes annotated by BLASTx with an E-value threshold of 10^−5^ against six protein databases. (**B**): E-value distribution of the top BLASTx hits against the NR database for each unigene. (**C**): Number and percentage of unigenes matching the top 24 species using BLASTx in the NR database.

**Figure 3:**
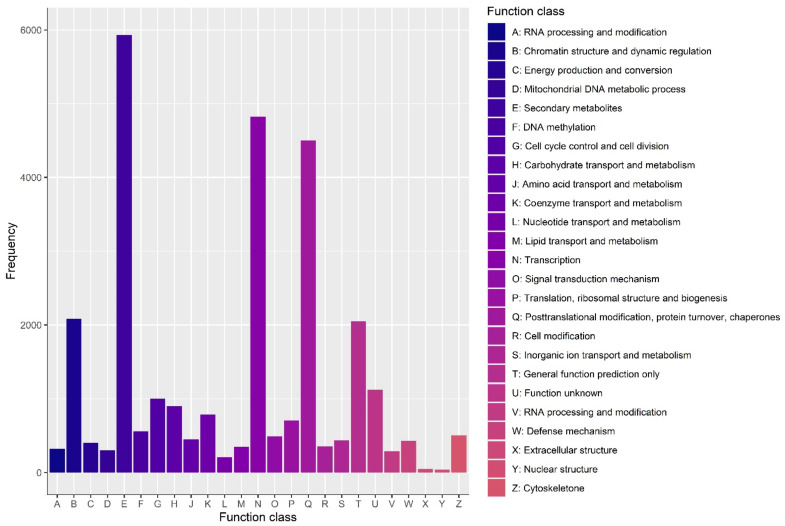
KOG categories of the annotated unigenes. The x-axis represents the names of the 25 groups, and the y-axis corresponds to the percentage of the number of genes in the group out of the total number of annotated genes (https://www.ncbi.nlm.nih.gov/COG/ (accessed on 10 February 2022), E values cutoff 1 × 10^−3^).

**Figure 4:**
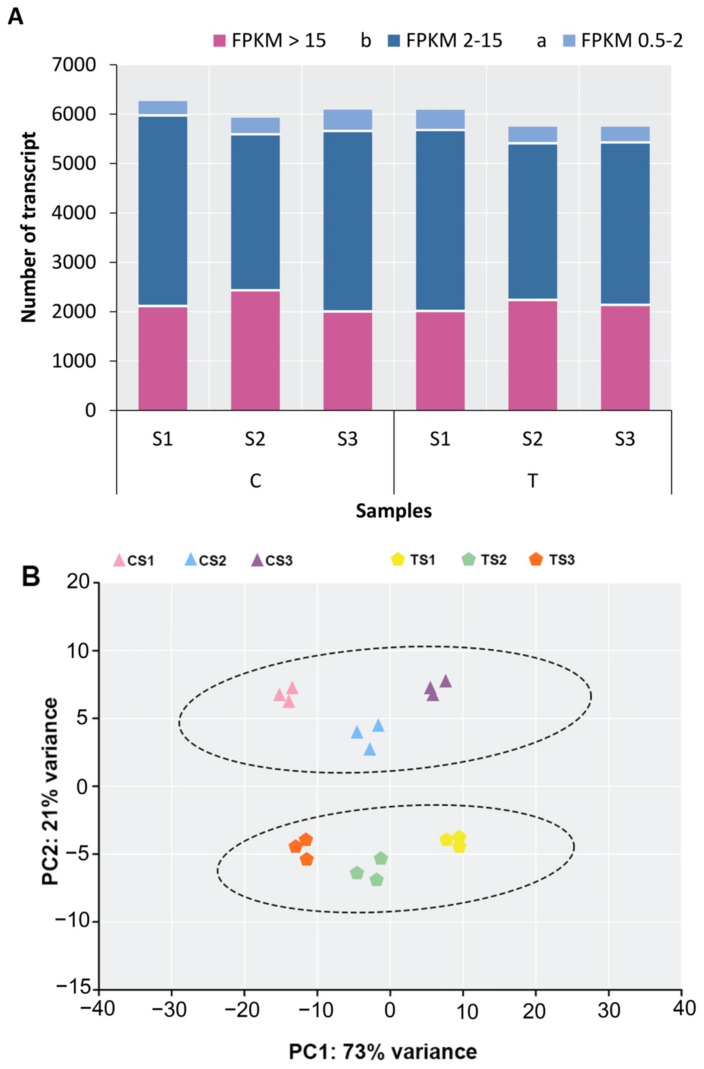
Statistics of global gene expression at different stages. (**A**): Number of detected transcripts in each sample. (**B**): Visualisation of principal component analysis (PCA) of all 18 samples in a 2D space. PCA was applied to Log2-transformed FPKM. Colour and shape indicate genotype and condition: CS1, stage (I) in control condition; CS2, stage (II) in control condition; CS3, stage (III) in control condition; TS1, stage (I) in treated condition; TS2, stage (II) in treated condition; TS3, stage (III) in treated condition.

The assembled unigenes were examined using eight public databases (i.e., NR, egg NOG, KOG, KEGG COG, GO, Swiss-Prot, and Pfam), with an E-value cut-off that was more than 1 × 10^−5^, to functionally annotate the transcriptome. Using this method, the annotation of 76.45% of the total unigenes (142,561) was performed. Among them, 128,376 unigenes (90.05% of all the unigenes annotated) could be annotated to the NCBI NR database, while the annotation of 93,192 (65.37%), 86,306 (60.54%), and 78,993 (55.41%) unigenes could be performed to the Swiss-Prot, Pfam, and KOG databases, respectively. The annotation of 41,128 (28.85%), 93,306 (65.45%), and 17,435 (12.23%) unigenes to the COG, GO, and KEGG databases was performed, respectively (Figure 2A). According to the statistical examination of the E-value features distributed in the Nr annotation, 88% of the assigned sequences exhibited strong homology (E-value < 10^−100^) and 12% had exceptionally strong homology (E-value 100^−150^) to the identified plant sequences (Figure 2B). Figure 2C shows the distribution of the top 24 species for the best match from each sequence. Blast2GO software was used to categorize 128,376 unigenes into 43 functional categories based on Nr annotation, with 18 GO terms relating to biological processes, 12 to cellular components, and 13 to molecular functions (Figure S1). KOG analysis was employed for analysing orthologous categorisation and the evolutionary rates of genes. The results showed that 78,993 unigenes (55.41% of all annotated unigenes) aligned with 25 KOG classifications (E-value cutoff 1 × 10^−3^). Among the different categories, a considerable number of unigenes were involved in clusters for the biosynthesis, transport, and catabolism of secondary metabolites (48.25%), followed by transcription (16.45%), protein turnover, posttranslational modification, chaperones (10.22%), and chromatin structure and dynamic regulation (8.10%). Only small proportions (less than 1%) of unigenes were assigned to extracellular structure, uncertain function, and cell modification. There were also higher proportions of genes associated with translation, ribosomal structure and biogenesis (7.05%), signal transduction mechanism (5.92%), DNA methylation (5.94%), and the mitochondrial DNA metabolic process (4.22%) (Figure 3). Transcripts with normalized read counts less than 0.5 FPKM were excluded from the study. CS1, CS2, and CS3 were discovered to express 28,365, 28,242, and 28,088 unigenes, respectively. Likewise, 27,810, 27,726, and 27,711 unigenes were found in treated samples from the different sepal maturation phases. Figure 4A shows the number of expressed transcripts dispersed in the 0.5–1 FPKM, 1–10 FPKM, and 10 FPKM ranges. The gene expression level correlation coefficient between the three biological replicate samples was greater than 0.73. The results of principal component analysis (PCA) indicated that the classification of the 18 samples could be easily classified into six groups: CS1, CS2, CS3, TS1, TS2, and TS3 (Figure 4B). The control and treated samples of the same developmental stage showed a distant clustering relationship, indicating that there was a clear distinction between the whole transcriptome profile of control and treatment at each developmental stage (Figure 4B).

### 2.4. DEG Identification and Functional Enrichment Analysis

**Figure 5:**
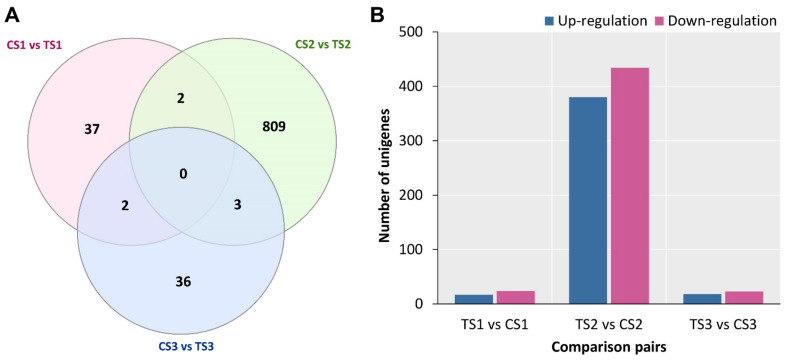
(**A**) The differentially expressed genes (DEGs) studied using RNA-Seq analysis during the colour development stages of *Hydrangea macrophylla*. (**A**) Venn diagram of the DEGs in three comparisons (CS1 vs. TS1, CS2 vs. TS2, and CS3 vs. TS3, respectively). (**B**) Vertical bar graph represents the number of up- (Blue boxes) and down- (purple boxes) regulated DEGs (FDR adjusted *p*-value cut-off of ≤ 0.05) based on their fold changes between the different stages.

The variations in gene expression were examined with the comparison of the three different sepal maturation stages, using thresholds of more than log2 (fold change) ≥2 and adjusted *p*-value less than 0.05 [33]. This resulted in a total of 896 DEGs (i.e., TS1 vs. CS1, TS2 vs. CS2, and TS3 vs. CS3, Figure 5A). TS2 vs. CS2 (814 DEGs) had the most DEGs among the three comparisons, with 380 and 434 unigenes up- and down-regulated, respectively. In contrast, TS3 vs. CS3 (41) had the fewest DEGs, with 18 and 23 unigenes up- and down-regulated, respectively (Figure 5B). Among all identified DEGs, the assignment of the 621 DEGs was made to one or more GO terms, and these DEGs revealed information about the molecular events that occur during the development of sepal, particularly colour formation. All DEGs were mapped to the GO database using the TopGO software (v2.12.0) to find highly enriched terms when related to the genomic background, using a corrected *p*-value of 0.01 (Fisher’s exact test) as the cutoff value. Among the three Gene Ontology categories, there were a total of ten enrichment GO keywords (Table 2). Under the biological process group, the most notable enrichment GO terms were pigment biosynthetic process, metabolic process, L-phenylalanine catabolic process, anthocyanin-containing, flavonoid biosynthetic process, response to abiotic stimulus, and pattern specification process. In the molecular function, catalytic activity, oxidoreductase activity, transporter activity, binding, peroxidase activity, electron carrier activity, and transcription factor activity were the most enriched whereas, in the cellular component (CC), intracellular and organelle cells were the most enriched.

**Figure 6:**
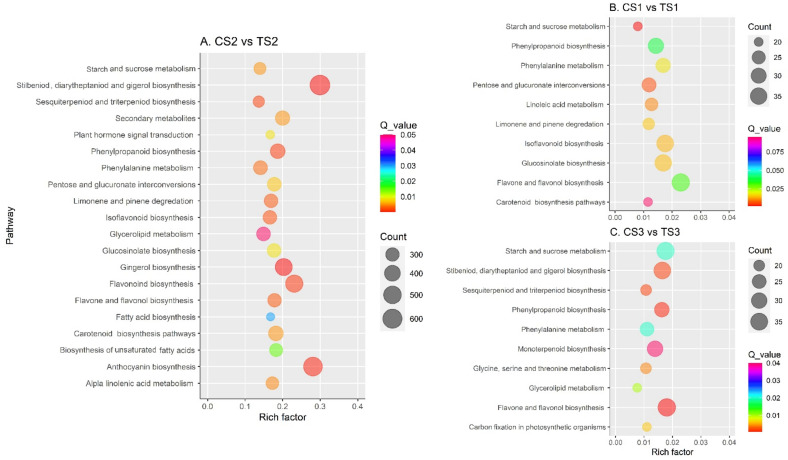
The scatter plots of the significant KEGG enrichment of functional pathway analysis of DEGs. (**A**) Top 20 pathway enrichment results for the DEGs of *Hydrangea macrophylla* at the CS2 vs. TS2 developmental stages. The x-axis indicates the log (corrected *p*-value). The greater the x-value, the smaller the corrected *p*-value, suggesting the enrichment is more significant. The y-axis represents the function descriptions of the enriched pathways. The anthocyanin biosynthesis pathway is the most significant enriched pathway. (**B**) Top 10 pathway enrichment results for the DEGs of *Hydrangea macrophylla* at the CS1 vs. TS1 developmental stages. The flavone and flavonol biosynthesis pathways are the most significant enriched pathways. (**C**) Top 10 pathway enrichment results for the DEGs of *Hydrangea macrophylla* at the CS3 vs. TS3 developmental stages. The flavone and flavonol biosynthesis pathways are the most significant enriched pathways, followed by starch and sucrose metabolism.

Understanding the relationship between the biological processes and the genes can be aided by pathway analysis. In TS1 vs. CS1, TS2 vs. CS2, and TS3 vs. CS3, the number of DEGs enriched among KEGG pathways was 26, 127, and 34, respectively, which were assigned to 15, 57, and 12 metabolic pathways, respectively. The most enriched 20 metabolic pathways were explored (Figure 6). In the comparison between TS1 and CS1, the biosynthesis of flavones, flavonols, isoflavonoids, and glucosinolates were enriched in TS1. In the comparison between TS2 and CS2, the biosynthesis of flavonoids, anthocyanins, stilbenoids, diarylheptanoids, and gingerol, as well as the biosynthesis of secondary metabolites, were increased in TS2. The biosynthesis of the metabolites flavone, flavonol, and phenylalanine was always significantly different at different developmental stages. The biosynthesis of flavonoids is predominant in the developmental stage S1 (CS1 and TS1), whereas the biosynthesis of anthocyanins was predominant in the developmental stage S2 (CS2 and TS2). Flavonoid biosynthesis was significantly enriched in TS1 compared with CS1, whereas anthocyanin was more enriched in TS2 compared with CS2. Al^3+^ treatment could induce anthocyanin biosynthesis rather than flavonoid biosynthesis to produce the blue colouration in hydrangea flowers. The carotenoid and isoflavone biosynthesis pathways, the other two metabolic processes involved in the floral colour formation, were also detected in the KEGG enrichment pathway. From these metabolic pathways, information about the pigment metabolism at three different colouring stages of *Hydrangea macrophylla* can be gleaned. The blue hue of Al^3+^-treated flowers may be closely associated with these metabolic pathways.

### 2.5. Identification of TFs and Establishment of Gene Co-Expression Network Analysis (WGCNA)

**Figure 7:**
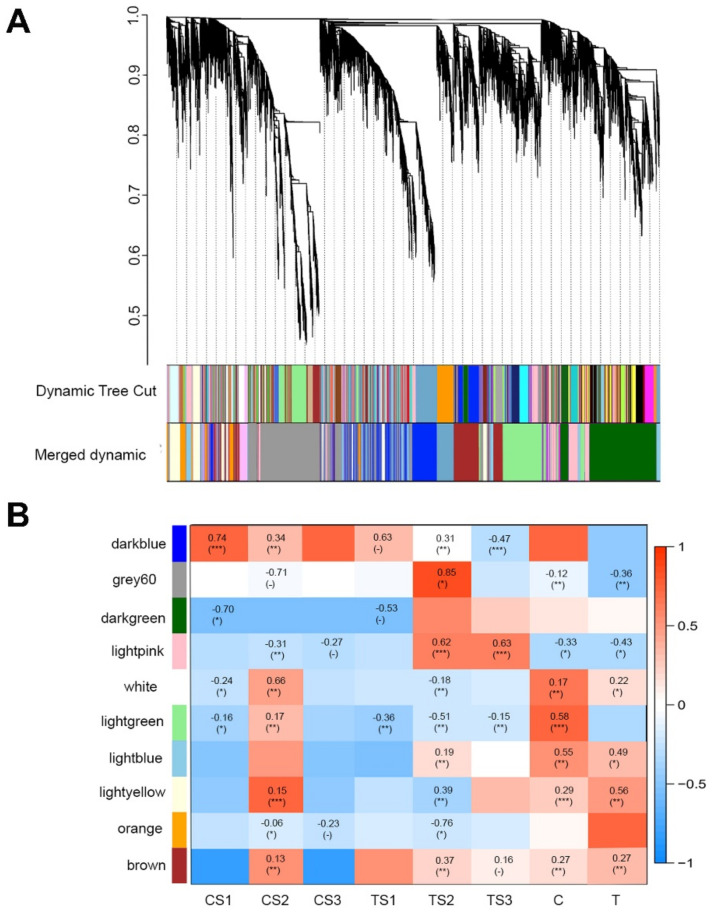
Gene co-expression modules discovered by WGCNA. (**A**) Clustering dendrogram of genes. Gene clustering tree (dendrogram) obtained by hierarchical clustering of adjacency-based dissimilarity. The coloured row below the dendrogram indicates the module membership determined with the dynamic tree cut method, along with the assigned colours of the merged modules and the colours of the original modules. (**B**) Module-trait associations using WGCNA. Factors of interest (x-axis) were correlated with each module (y-axis). The first value in each square is the correlation and the second value in parentheses is the *p*-value of the association. The more positively correlated the module and factor, the redder the square; the more negatively correlated, the bluer. The coding of the factors was as follows: CS1, CS2, CS3, TS1, TS2, TS3, C, and T. * Significant at *p* < 0.05; ** Significant at *p* < 0.01; *** Significant at *p* < 0.001, (-) means no content.

Using BLASTX (E-values cutoff 1 × 10^−5^), the assembled unigenes were aligned with the plant transcription factor database (PlantTFDB) and a total of 88 TFs from eight TF families were found. The MYB TF family had the most members (25), followed by the WD40 (20 TFs), HD-ZIP (14 TFs), bHLH (11 TFs), C2H2 (10 TFs), AP2-ERF (5 TFs), and NAC (3 TFs) families (Table S3). WGCNA was used to construct co-expression gene network modules to further investigate potential unigenes associated with pigmentation transition during the successive developmental stages of the two experimental conditions (Figure 7A, note: in the dendrogram, each module is represented by a branch, while each gene is shown as a leaf). The co-expression network constructed using the 621 DEGs that remained after eliminating the low-expression unigenes from the total 896 DEGs was integrated into 10 modules. The largest of which was the light blue module with 345 unigenes, and the smallest contained only 26 unigenes (dark green). Figure 7B shows the unigene distribution in each module (indicated with different colours) and the connections between module and trait. The 9 out of 11 DEGs associated with anthocyanin biosynthesis and 2 of 12 DEGs related to flavonoid metabolism are included in the brown module. This indicates that the brown module unigenes play a key role in anthocyanin and flavonoid metabolism. We were particularly interested in the modules that were enriched in the control or treatment groups, especially blue and pink in S2, which aid in distinguishing the flower colour phenotype caused by an environmental pollutant. The modules of interest were therefore selected based on |*r*| > 0.5 and *p* ≤ 0.05 criteria and then annotated using KEGG and GO analysis. The light green module was closely associated with TS2. Many colour formation pathways were enriched in the light green module (*p* ≤ 0.01). The three major metabolic pathways were phenylpropanoid biosynthesis (ko00940, 30 DEGs), anthocyanin biosynthesis (ko00942, 19 DEGs), and flavonoid biosynthesis (ko00941, 10 DEGs) (Table S4). Pearson correlation coefficients of structural genes and transcription factors were determined with SPSS version 17.0 based on their FPKM values. The FPKM values of the unigenes of the transcription factor *HymMYB2* and the two *WDR40* unigenes were positively (*p* ≤ 0.01) and negatively (*p* ≤ 0.01) correlated with the values of *CYP73A*, *F3′5′H*, *C3H*, *C2H2*, *DFR*, and *ANS* FPKM, respectively. In addition, the FPKM level of a *WDR68* unigene was correlated negatively with the levels of *DFR* and *F3H* (*p* ≤ 0.01) (Table S5). The transcription factor *HymMYB2*, together with other transcription factors such as WER-like and *WDR40*, might play an important role in the formation of the blue colour of infertile hydrangea flowers.

### 2.6. Candidates Accountable for the Gain of Blue Colour in Hydrangea with Pink-Coloured Flower

Table 3 shows the expression patterns of 15 potential genes based on closed modules. In summary, in the treated plants, all five PALs were down-regulated during sepal maturation, whereas in the untreated plants, they initially remained constant or decreased and later increased. Moreover, their relative expression levels were significantly higher in treated individuals S1-S3 than in the untreated groups. *PAL9* and *PAL6* were found to be putative hub genes for the dark green module. *4CL12* and *4CL14* were shown to be enriched in the dark green module, and *4CL12* was recognized as a possible hub gene for this module. The significantly higher expression of the *4CL12* gene in S1–S3 of the treated plants, when compared to the untreated groups, indicated that *4CL18* had a crucial role in the signalling pathway. The orange module had three enriched *CHSs*, with *CHS2* and *CHS4* recognized as possible hub genes that showed similar changes in expression during different phases in the treated and untreated groups. The relative expression levels of *CHS1* and *CHS3* in CS3 were 2.3 and 1.5-fold higher than in TS3, respectively. We also searched for two *CHRs*, which were enriched in these critical modules and discovered that the change in the expression patterns of *CHR1* and *CHR3* were compatible with the enriched *CHSs*. In addition, these modules enriched *F3′H4*, *F3′H3*, *F3′H2*, *F3′5′H*, *FLS1*, *FLS2*, *PIP2*, *TIP1*, *UA3GT*, *PAP2*, *DFR1*, *DFR2*, *CYP75A*, and *CYP75B1*. The expression levels of *F3′5′H* were 2.1 and 3.2 times higher in treated plants than in untreated plants in S1 and S2, respectively. *DFR1* was up-regulated and peaked in S3 during flower development in Al^3+^-treated plants, whereas it was virtually absent in untreated plants. There was an up-regulation in the *DFR2* gene in treated plants and it peaked at S3, whereas in untreated plants its expression was low and remained stable. The expression levels of *DFR1* and *DFR2* were significantly higher in all stages of the treated plants than in the untreated plants. The expression levels of *CYP75A* and *CYP75B1* were also higher in the treated plants (Table 3). 

### 2.7. qRT–PCR of the Transcriptomic Data

**Figure 8:**
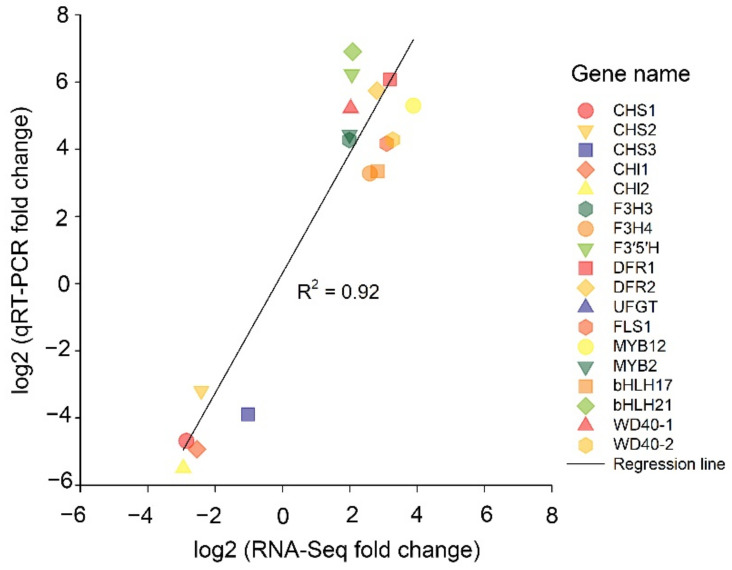
Correlation analysis between qRT-PCR and RNA-Seq results for *CHS1*, *CHS2*, *CHS3*, *CHI1*, *CHI2*, *F3H3*, *F3H4*, *F35H*, *DFR1*, *DFR2*, *UFGT*, *FLS1*, *MYB12*, *MYB2*, *Bhlh17*, *Bhlh21*, *WD40-1*, and *WD40-2* in *Hydrangea macrophylla*.

For the validation of the transcriptome sequencing data, the sequences of 15 nuclear unigenes related to the formation of the colour blue, which showed varying expression levels in the two experimental groups, were subjected to quantitative real-time PCR using the designed primers. The results show that the expression levels of transcriptome and qRT–PCR analyses are significantly correlated with each other with a correlation coefficient of R^2^ = 0.92, indicating that the genes studied are involved in the signalling and/or metabolic pathways associated with colour formation (Figure 8). 

## 3. Discussions

Flower colour has long fascinated scientists and breeders, and it has been demonstrated that flower colour develops as a consequence of interactions between genes and external environmental conditions [34]. Consequently, the development of the colour blue in the *H. macrophylla* cultivars can be attained by changing the growing conditions. For instance, altering the pH of the soil or the addition of exogenous Al^3+^ can cause colour variation in some barren flowers of *H. macrophylla*. However, the knowledge about the underlying process of colour change and tolerance to acidic pH is still limited. Breeding plants that can tolerate acidic soils is a pressing issue in agricultural and plant physiology research. In this competition, Chen et al. performed a genome-wide transcriptome analysis of Al response genes in hydrangea roots and leaves using an RNA-Sequencing approach. Numerous transporters were involved in the transportation of the Al–citrate complex from hydrangea roots, including those from the MATE and ABC families. The aluminum transporter Nramp, a plasma membrane transporter for Al uptake, was upregulated in roots and leaves under Al stress, suggesting that it may play an important role in Al tolerance by lowering toxic Al levels. However, the signalling pathways and potential genes involved in the colour change remain to be elucidated [29]. In the current study, next-generation sequencing technology was utilized to compare the transcriptomes of hydrangea sepals grown under Al^3+^-treated and control conditions at three sepal maturation stages to discover the genes and signaling pathways responsible for the colour change in response to Al hyper accumulation.

### 3.1. Comparison of Genes Involved in Flavonoid Biosynthesis in Hydrangeas Grown under Different pH Conditions

Flavonoids are vital pigments found in many plant sepals [35]. Anthocyanins are the end products of the biosynthetic pathways of flavonoids. They produce a wide variety of colours, from pale yellow to blue–violet [36]. The accumulation of the floral anthocyanins malvidin and petunidin causes the colour difference between Al^3+^-treated (blue–violet) and untreated (pink) hydrangea sepals (Figure 1). The conversion from pink to blue needs a change in the pathway of anthocyanin biosynthesis, which most likely occurs several processes before the production of petunidin and malvidin. Thus, the abundance of potential genes involved in flavonoid biosynthesis was evaluated to find vital genes involved in blue colour metabolism. Several isoforms of flavonoid synthesis, including *4CLs*, *PALs*, *CHIs*, *CYP73A*, *DFRs*, *ANSs*, *F3H*, *F3′5′H*, and *UA3GT*, showed distinct expression patterns in the Al^3+^-treated plants with blue–purple flowers compared with untreated plants with pink flowers, suggesting that the alteration in the expression of these genes, induced exogenously, may occur much earlier than the phenotypic changes. *CHS*, *CYP73A*, and *CHI* are upstream genes of the flavonoid biosynthetic pathway, whereas *ANS*, *F3H*, *F3′5′H*, *C3′5′H*, *DFR*, *and CYP75B1* are downstream genes. They encode important enzymes in the biosynthetic pathway of flavonoids and thus contribute to the formation of flower colour [37]. The effects of *CHI* and *CHS* on flavonoid accumulation are considerable. The initial reaction step in the flavonoid biosynthetic pathway is catalysed by *CHS* contributing to the formation of the intermediate product chalcone, which is required for all flavonoid classes [38]. In overexpressed *CHI* tobacco plants, flavonoids were increased but anthocyanins were not detectable [39]. The overexpression of the peony-derived *CHI* gene in tobacco also increased flavonoid accumulation [40]. Almost all unigenes expressing *CHI* and *CHS* were up-regulated at S1 during the development of infertile flowers in both experimental groups, and flavonoid concentration was maximal at this time. When the early expressed genes in the flavonoid biosynthesis pathway were over-expressed, it resulted in flavonoid accumulation and provided precursors for anthocyanin biosynthesis [41]. The biosynthesis pathway of flavonoids is dependent on *F3H*, *F3′H*, and *F3′5′H*. They catalyse the hydroxylation of flavonoids required for anthocyanin production, such as dihydrokaempferol, dihydromyricetin, and dihydroquercetin [42,43]. *F3H* catalyses the conversion of naringenin to dihydroflavones. In the presence of NADPH and *DFR*, the three forms of dihydroflavones are reduced. It has been observed that flower colour can vary greatly depending on the type and extent of *DFR* expression and that *DFR* is most strongly expressed in sepals with a high concentration of anthocyanins [44]. We have discovered two different *DFR* copies. *DFR-2* is predominantly expressed in pink flowers (it is almost undetectable in blue samples) and has an N residue in the substrate specificity region at the third position that has been shown to cause substrate affinity for the pelargonidin-like precursor, dihydrokaempferol, in a variety of angiosperms [45]. *DFR-1*, on the other hand, is mainly produced in blue flowering plants and mainly has a D residue at this position, which confers lower or no substrate affinity for dihydrokaempferol in other species [46]. Moreover, NADPH cytochrome P450 expression was significantly higher in the treated plants than in the control group during the first and second phases of sepal maturation. NADPH cytochrome P450 reductase was shown to catalyse electron transfer from NADPH to *F3′5′H* in petunia, resulting in the blue–purple colour of the plant [47]. The hydroxylation of dihydrokaempferol is catalysed by *F3′5′H* and leads to the formation of a delphinidin precursor [48]. The loss of *F3′5′H* function in *Antirrhinum* spp. [49] or reduced *F3′5′H* expression in *Phlox drummondii* [50] results in a transition from blue to red colour. *C3′5′H* is an enzyme important for the production of delphinidin and promotes blue flower formation [51]. *ANS*, a vital enzyme that catalyses the last step of the flavonoid biosynthetic pathway, can also catalyse the conversion of proanthocyanidins into coloured anthocyanins. Through the induction of anthocyanin synthesis in sepals by Antirrhinum majus dihydroflavonol 4-reductase (*AmDFR*) and anthocyanidin synthase (*MiANS*) genes (*AmDFR*), transformed through a sequential Agrobacterium-mediated transformation, the flower colour of forsythia (Forsythia x intermedia cv ‘Spring Glory’) was altered [52]. In the current study, the downstream genes *F3′H*, *F3′5′H*, *C3′5′H*, *CYP75B1*, *DFR1*, and *ANS* of the flavonoid biosynthesis pathway were all increased at the first and second developmental stages of Al^3+^-treated plants. We also analysed the expression levels and patterns of all glycosyltransferases of the anthocyanin biosynthetic pathway and observed four *UA3′GT* homologous unigenes. However, their FPKMs were extremely low, and there were no significant differences in the expression levels at the three sepal maturation stages of the treated plants, but there was a significant difference in their expression at the second stage between the treated and control groups, indicating that *UA3′GTs* may be one of the key enzymes for the accumulation of delphinidin-3′-glycosides, the main anthocyanins in blue sepals. Kogawa et al. discovered that *UA3′5′GT* is critical for the accumulation of polyacylated anthocyanins, called ternatins, in *Clitoria ternatea*. *UA3′5′GT* could be glycosylated at the 3′, 5′ positions of delphinidin [53]. The blue–violet flower colour of treated individuals suggests that the basic regulation of gene transcription from upstream *ANS* (anthocyanidin synthase), *F3′5′H* (flavonoid 3′,5′-hydroxylase) and *DFR* (flavanone 4-reductase) may play a vital role in the buildup of flavonoid intermediates and the transition of flower colour.

### 3.2. Identification of Hub Genes Related to Flower Formation by WGCNA

Understanding the changes in blue flower phenotype caused by external influences of the wild type (with pink colour) could shed light on the mechanisms of flower colouration in hydrangea. Any functional changes in critical enzymes of the flavonoid biosynthetic pathway, including changes in the frequency of gene transcription and branching changes of flavone products, could lead to a repeated transition from blue to red/pink [54]. The most important finding of this work was that, by using WGNCA, we were able to identify Al^3+^ treatment-specific gene modules (Figure 7B). This showed that 2 *DFRs*, 2 *4CLs*, *3 CHRs*, *9 PALs*, *4 CHSs*, *F3′H4*, *2 UFGTs* and *MYB* were strongly related with modules relevant to the TS2 or treatment group. They all showed significant variation in transcript levels between treated and untreated individuals, demonstrating that they play a crucial role in floral variation. It is important to note that the above genes were not the ones with the highest expression, suggesting that the genes with the highest expression are not essential for flower colour differentiation [55]. Thus, the usage of WGCNA analyses in this work delivered a good method for identifying key genes associated with different developmental conditions. Wang et al., 2020 used WGCNA analysis to identify nodule genes involved in heavy metal transport, which were found to be particularly abundant in nodules [56]. In camellia, a similar WGCNA approach was used to reveal unigenes related to flower colour, and it was shown that *CHS*, *F3H*, *ANS*, and *FLS* have a crucial role in controlling the synthesis of flavonols and anthocyanidins [57]. Tan et al. [58] further used WGCNA to extract the Cd-coupled co-expression gene modules from the 22,080 transcripts in 17 RNA-Seq datasets and recognized 271 transcripts as universal Cd-regulated DEGs, which are key components of the Cd-coupled co-expression module. The four hub genes were found upstream of the flavonoid biosynthetic pathway, suggesting that blue flower colouration was mainly stimulated upstream in the treated plant. The reduced expression of *4CL8*, *PAL9*, and *PAL6* in both treated and untreated plants is consistent with the results of Wang et al. [59]. The decreased expression of *4CLs* and PALs altered the level of cinnamic acid in the ripe fruit peel, according to these researchers [59]. We hypothesized that the reduced expression of *4CL8*, *PAL6*, and *PAL9* would affect cinnamic acid concentration in sepals from both treated and untreated plants. The increased expression of *CHIs* and *CHSs* in TS2 may play a vital part in the biosynthesis of other flavones, such as isoflavones, which contribute to the colour of many hydrangea flowers.

### 3.3. Identification of Transcription Factors Related to Flower Colour Transition 

**Figure 9:**
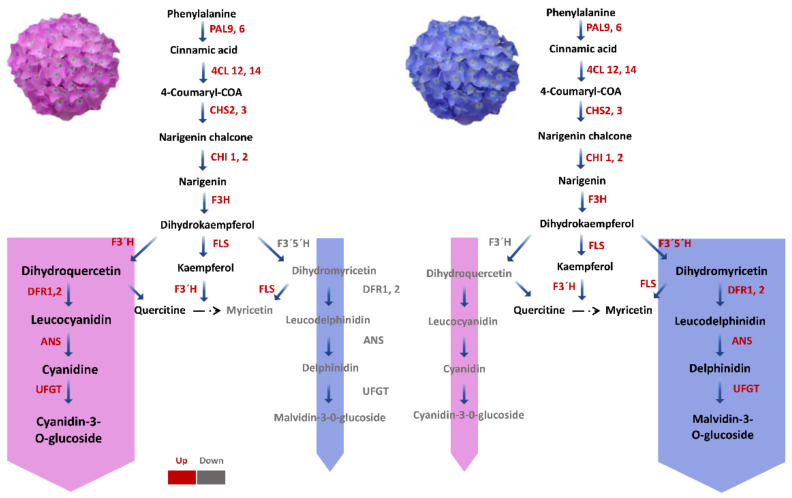
Key pathways for *Hydrangea macrophylla* flower expressions. Flavonoid biosynthetic pathways are shown in treated and untreated hydrangea. Expression of *F3′5′H*, *F3′H*, and *F3H/FLS* transcripts may lead to an increase in kaempferol and myricetin, further reducing anthocyanin production. In Al^3+^-treated plants, *DFR* expression combined with UFGT availability may stimulate anthocyanin synthesis, resulting in the formation of a blue colour. Phenylalanine ammonia lyase (*PAL*), 4-coumarate CoA ligase (*4CL*), chalcone synthase (*CHS*), chalcone isomerase (*CHI*), flavanone 3-hydroxylase (*F3H*), flavonoid 3′,5′-hydroxylase (*F3′5′H*), dihydroflavonol 4-reductase (*DFR*), anthocyanin synthase (*ANS*). Blue and purple boxes mark anthocyanin compounds present in each flower colour.

*MYB*, *WDR*, and *bHLH* transcription factors control the flavonoid biosynthetic pathway in several higher plant species [60,61]. Transcription factors can regulate structural genes either alone or in cooperation, and they can be positively or negatively regulated. Generally, transcription factors affect flower colour in different ways. The MYB–bHLH–WD40 (*MBW*) ternary transcription complex triggers numerous late flavonoid biosynthesis genes (LBGs), which include three regulatory protein classes, including *bHLHs*, *R2R3*-*MYBs*, and TRANSPARENT TESTA GLABROUS1 (*TTG1*; also known as *WD40*) [62,63]. *MYB* transcription factors, *bHLHs*, and *WD40* regulate the expression of *ANS* and other downstream genes in Arabidopsis and affect anthocyanin biosynthesis [59]. *LrMYB15*, a transcription factor regulating *DFR*, *CHSa*, *ANS*, and *CHSb*, was observed to be involved in anthocyanin biosynthesis in lilies [64]. MYBs were the largest TF family in our analysis with 23 genes. Analysing DEGs and transcription factors based on their co-expression pattern revealed that among these *MYB*s, the change of flower colour was consistent with that of the expression profiles of 17 genes, with only 5 genes showing an opposite expression trend. The gene *HymMYB2* was expressed at all developmental stages of Al^3+^-treated plant sepals. TS2 showed the highest expression level. Despite previous findings of a connection between the expression of *HymMYB2* and the total amount of anthocyanins in sterile flowers of numerous hydrangea varieties, no such association was found in this study [51], and the expression pattern in our investigation was not the same between treated and untreated plants. The expression of *HymMYB2* was significantly elevated in the treated groups, but it was nearly undetectable in the control plants. This shows that *HymMYB2* regulates anthocyanin intermediates in hydrangea with some specificity. The level of *HymMYB2* expression was highly associated with the expression of important genes *DFR*, *F3H*, *ANS*, *C3′5′H*, and *WDR40*, according to co-expression analysis. The transcription factor *HymMYB2* may act on *C3′5′H*, *ANS*, and *DFR* in the biosynthetic pathway of anthocyanin in hydrangea, which is similar to the function of the *PeMYB11*, *PeMYB12*, and *PeMYB2* genes (structural genes) in Phalaenopsis [65]. Based on the data presented above, different flavonoid biosynthetic pathways were determined in treated and untreated hydrangeas (Figure 9). In summary, flavonoid production in Al^3+^-treated plants is advanced by *PAL* and *4CL* compared with untreated specimens, whereupon a branch of isoflavone biosynthesis, regulated by *CHS* and *CHR*, completes the anthocyanin synthesis pathway. In addition, the elevated expression of *F3′5′H*, *F3′H* and *F3H/FLS* leads to an increase in other flavonoid molecules, such as kaempferol and myricetin, which further decreases anthocyanin production. Lastly, a high *DFR* expression combined with the availability of *UFGT* may stimulate the synthesis of anthocyanin, leading to blue colour formation. 

## 4. Materials and Methods

### 4.1. Plant Material

*Hydrangea macrophylla* ssp. *serrata* were provided by the Koetterheinrich breeding company (Lengerich, Germany) at week 48 and placed in a greenhouse at IPK Gatersleben under the condition of an 18-h photoperiod with 200 µmol m^−2^ s^−1^ light intensity, a temperature of 21/19 °C (day/night), and 60% relative humidity. Plants were divided into two groups, either as the control without additional treatment or with external Al^3+^ application. All plants were supplied with 1 g Universal Weiβ fertilizer (with the analysis of 15 + 0 + 19) per liter of irrigation water in weekly intervals. For the treated group, 1 g of aluminum sulfate (Al_2_ (SO_4_)^3−^) was added to this solution. Aluminum-treated groups and control groups were arranged in a block with complete randomisation and three replicates per group. For each of the developmental periods, three experimental samples were harvested from cuttings obtained from the same plant at the early blooming stage: pale yellow, middle blooming stage: light blue (Al-treated group) to light pink (control group), and full blooming stage: dark blue (Al-treated group) to dark pink (control group). The pH was recorded once during all cycles in the substratum of all the plants with the dilution method 1:2 reported by David et al. [66]. Healthy, barren flowers with appropriate development and no visible diseases or pests were procured, rinsed thrice with deionized water to prevent contamination during sampling, then immediately immersed in liquid nitrogen and placed in a refrigerator at −80 °C [67]. 

### 4.2. RNA Extraction, cDNA Library Creation, and Sequencing

The RNeasy Plant Mini Kit (Qiagen, CA, USA) was used for total RNA extraction following the manufacturer’s instructions. The TURBO DNA-free^TM^ kit was used to purify RNA (Invitrogen, CA, USA). The Agilent 2100 Bioanalyzer (Agilent Technologies, CA, USA) was employed for quality assessment and to measure the concentration of the extracted RNA. For all samples, the RNA integrity number (RIN) was more than eight. A total of 18 RNA-Seq libraries (including three biological replicates at three stages for treated (TS1, TS2, and TS3) and control samples (CS1, CS2, and CS3) were constructed from about 2 µg of hydrangea sepals as per the manufacturer’s protocol (Lexogen GmbH, Vienna, Austria). The libraries were pooled in an equimolar way and the Agilent 4200 Tape Station System (Agilent Technologies, Inc., Santa Clara, CA, USA) was used for electrophoretic analysis. Using the Illumina HiSeq2500 equipment, libraries were quantified and sequenced (paired-end sequencing, rapid run, 2 × 101 cycles, onboard clustering) (Illumina, San Diego, CA, USA).

### 4.3. Data Analysis

Firstly, clean reads were obtained by eliminating the low-quality sequences, adapter sequences, unknown reads (N percentage >10%), and ribosome RNA from the raw reads. Later, these clean reads were subjected to de novo transcriptome assembly via the trinity platform (http://trinityrnaseq.github.io/ (accessed on 10 February 2022)) without digital normalisation, applying min_kmer_cov = 2 and other default parameters [68,69]. Longer contigs were constructed from short reads with overlap areas and then joined to procure transcripts (removing those smaller than 200 bp) and grouped depending on the nucleotide sequence identity. To prevent redundant sequences, the longest transcripts in the cluster units were classified as unigenes. The BLASTx alignment was used to annotate unigenes (E-value ≤ 10^−5^) in various protein databases, including the Swiss-Prot protein database (http://www.uniprot.org/ (accessed on 8 February 2022)), the NCBI non-redundant (NR) protein database (ftp://ftp.ncbi.nih.gov/blast/db/ (accessed on 10 February 2022)), the Kyoto Encyclopedia of Genes and Genomes (KEGG) pathway database (http://www.genome.jp/kegg/ (accessed on 10 February 2022)), the Clusters of Orthologous Groups of proteins (COG) database (http://www.ncbi.nlm.nih.gov/COG/ (accessed on 10 February 2022)), the eukaryotic Orthologous Groups of proteins (KOG) database (http://www.ncbi.nlm.nih.gov/KOG/ (accessed on 13 February 2022)), and the Gene Ontology (GO) database (http://www.geneontology.org/ (accessed on 3 March 2022)). After predicting the amino acid sequences of unigenes by GetOrf (EMBOSS: v 6.3.1), these sequences were aligned in the protein family (Pfam, http://pfam.xfam.org/ (accessed on 3 February 2022)) using the HMMER software suite (v3.0) (E-value ≤ 10^−10^). Out of the seven databases, only the best alignment results were selected to decide the unigene annotations. 

### 4.4. Expression Annotation

Bowtie (v4.4.7) alignment package was used to trace reads to the unigenes. Based on the comparison results, RSEM (RNA-Seq by Expectation maximisation) was used to estimate the expression levels [70]. The Fragments Per Kilobase of transcript per Million mapped reads (FPKM) method was applied to represent the differences in the unigene expression among the samples [70]. Differential expression analysis between different experimental conditions was performed using the DESeq package (v1.18.0) [71]. The differentially expressed genes (DEGs) were identified using log2FC ≥ 2 and *p*-value ≤ 0.05 [24]. The iTAK software was used for the prediction of the plant transcription factors [72]. For identifying the transcription factors (TFs), all the annotated unigenes were compared against the plant transcription factor database (Plant TF DB v. 4.0), and the best hits in *Arabidopsis thaliana* were considered as TFs. Using the gene co-expression networks generated from the DEGs and TFs discovered, the transcriptional architecture of anthocyanin, carotenoid, and flavonoid biosynthesis were established by the WGCNA (weighted gene co-expression network analysis) program [73] and then visualized using Cytoscape v. 3.5.1 (San Diego, CA, USA) [26].

### 4.5. GO and KEGG Pathway Enrichment Analysis for Differentially Expressed Unigenes

GO and KEGG pathway enrichment analyses were performed for the differentially expressed unigenes. The topGo package (v2.12.0) was applied for enrichment and refinement of the collected GO annotation, using the “elim” approach and the Kolmogorov-Smirnov test. In-house scripts were used for KEGG pathway enrichment in accordance with Fisher’s exact test. Bonferroni correction was used to obtain enriched *p*-values. The corrected *p-*value of 0.05 was used as a criterion to determine whether gene sets were significantly enriched.

### 4.6. Quantitative Reverse Transcription–Polymerase Chain Reaction-Based Validation

Five unigenes related to the biosynthesis of anthocyanin and 10 unigenes associated with the biosynthesis of carotenoids were chosen for quantitative reverse transcription–polymerase chain reaction (qRT–PCR) analysis. For qRT–PCR, the TransStart Top Green qPCR SuperMix (TransGen Biotech, Beijing, China) and a Bio-Rad CFX96 RT-PCR system (Bio-Rad, Hercules, CA, USA) were used with the following reaction conditions: denaturation at 94 °C for 60 s and 45 cycles of amplification (94 °C for 5 s, 60 °C for 15 s, and 72 °C for 10 s). The 2^−ΔΔCt^ method was used for calculating the relative expression levels of target genes against the internal control [74]. To normalize the relative expression levels of target genes, the *H. macrophylla* actin gene was employed as a control [75]. Supplementary Table S1 lists the gene-specific primers. For each experiment, three biological and three technical replicates were used.

### 4.7. Estimation of Relative Pigment Content

The extraction of anthocyanin was carried out using fresh sepal tissue acquired from three sepal maturation phases in Al^3+^-treated and untreated samples. Together, 0.5 g of tissue from each sample was powdered using 1 mL of 98% methanol comprising 1.6% formic acid at 4 °C. Following ultrasonic extraction for 30 min, the samples were centrifuged at 12,000× *g* for 10 min, the supernatant was transferred to new tubes, and the residues were re-extracted. Later, the supernatants were pooled and filtered through 0.45 mm nylon filters (Millipore). Cyanidin 3-O-glucoside, delphinidin 3-O-glucoside, peonidin 3-O-glucoside, pelargonidin 3-O-glucoside, petunidin 3-O-glucoside, and malvidin 3-O-glucoside were among the standard compounds (ZZBIO Co., Ltd., Shanghai, China). An amount of 10 µL of the extract was analysed by HPLC according to the method of Zheng, et al. [76], (Rigol L-3000, Beijing, China). Three biological replicates yielded mean results and standard errors (SE). For flavonoid extraction, 200 mg of sepal tissue was pulverized with liquid nitrogen, extracted in 10 mL of methanol solution, incubated in dark conditions for 24 h at 4 °C, and then suspended by sonication for 1 h. After centrifugation at 10,000 rpm for 10 min, the supernatant was filtered through a 0.22 mm membrane filter. After removing 2 mL of the supernatant, 2 mL of a 1.5% AlCl_3_ solution and 3 mL of 1 M sodium acetate (pH 5.0) were supplemented, and ten minutes later, a UV-Vis spectrophotometer was used to measure the absorbance at 415 nm [77]. The trend in flavonoids’ relative content was observed across the three periods on the basis of absorbance values. For carotenoid analysis, liquid nitrogen was used to crush 200 mg of sepal tissue, which was then extracted in 10 mL petroleum ether under dark conditions at 4 °C for 24 h and suspended by sonication for 1 h. After centrifugation at 10,000 rpm for 10 min, the supernatant was collected and filtered through a 0.22 mm membrane filter. A UV-Vis spectrophotometer was used to measure the absorbance at 440 nm [78]. The absorbance readings were used to observe the trend in the relative number of carotenoids across the three periods.

### 4.8. Statistical Analyses

All measurements were executed in triplicate, and outcomes are expressed as mean and standard deviation (SD). The Statistical Package for the Social Sciences (SPSS) v. 19.0 software was used to perform statistical analyses wherein the mean values of each developmental stage were compared using a one-way analysis of variance (ANOVA) according to the Duncan multiple choice test at *p* < 0.05.

## 5. Summary

Transcriptome analysis was used to investigate whether and how the pathways of anthocyanin and flavonoid in Al^3+^-treated (blue–violet) and untreated hydrangea plants with pink flowers contribute to the formation of colour. The quantitative analysis of the essential anthocyanins in the flowers of Al^3+^-treated hydrangea were delphinidin, petunidin, and malvidin derivatives, which were absent in the untreated plants. Transcriptome analysis of sepals from two different growth conditions and three different stages of sepal maturation revealed 186,477 unigenes. Several genes that alter or inhibit flavonoid biosynthetic pathways, competing with the production of other flavonoids or altering the synthesis of anthocyanins, may partially be responsible for the blue colour phenotype in hydrangea flowers. *DFR* and *UFGT* are among key genes involved in the blue colouration. Al^3+^-treated plants produce more delphinidin derivatives and have a higher ratio of *F3′5′H/F3′H* transcription than the untreated pink plants. In addition, we also identified several TF families such as *WD40*, *bHLH17*, and *MYB11*, which are likely important regulators in anthocyanin biosynthesis, chlorophyll metabolism, and carotenoid biosynthesis. This study contributes to a better understanding of molecular mechanisms of colour formation in hydrangea that has scientific value and helps breeders design and adapt the desired flower colours.

## Figures and Tables

**Table 1:** Length-wise distribution of contigs and unigenes in the transcriptome of Hydrangea sepals.

Length Range	Contigs	Unigenes
200–300	120,191 (31.13%)	48,868 (26.20%)
300–500	92,335 (27%)	60,448 (32.41%)
500–1000	72,233 (21.11%)	48,027 (25.75%)
1000–2000+	57,309 (16.75%)	29,134 (15.62%)
Total number	342,068	186,477
Total length	845,487,394	94,130,265
N50 length	903	794
Mean length	624	618

**Table 2:** Functional enrichment analysis of DEGs in hydrangea sepal’s transcriptome using Gene Ontology.

Gene Ontology Term	Cluster Frequency	Genome Frequency	*p*-Value	Corrected *p*-Value
BP: Pigment biosynthetic process (GO:0046148)	31 out of 207, 14.97584%	24 out of 298, 8.05369%	6.61 × 10^−9^	0.000000
BP: Metabolic process (GO:0008152)	4 out of 207, 1.67687%	2 out of 298, 0.67111%	5.62 × 10^−5^	0.000622
BP: Developmental process (GO:0032502)	2 out of 207, 0.96618%	1 out of 298, 0.33557%	4.33 ×10^−5^	0.000008
BP: Anthocyanin process (GO:0046283)	3 out of 207, 1.44927%	2 out of 298, 0.671114%	3.45 × 10^−5^	0.000018
BP: Flavonoid biosynthetic process (GO: 0009813)	4 out of 207, 1.93236%	1 out of 298, 0.33557%	6.89 × 10^−5^	0.00173
BP: Chalcone biosynthetic process (GO:0016210)	6 out of 207, 2.89855%	3 out of 298, 1.00671%	5.22 × 10^−5^	0.000000
MF: Catalytic activity (GO:0003824)	3 out of 237, 1.26533%	1 out of 339, 0.29498%	7.86 × 10^−5^	0.004689
MF: Binding (GO:0005488)	6 out of 237, 2.53145%	3 out of 339, 0.83454%	4.34 × 10^−5^	0.00173
MF: Electron carrier activity (GO:0009055)	9 out of 237, 3.79746%	5 out of 339, 1.47579%	5.76 × 10^−4^	0.002882
MF: Transcription factor activity (GO:0003700)	10 out of 237, 4.219400%	6 out of 339, 1.7699%	2.14 × 10^−4^	0.005734
CC: Intracellular (GO:0005622)	5 out of 177 2.82475%	1 out of 259 0.38610%	3.65 × 10^−5^	0.009393
CC: Organelle (GO:0020037)	3 out of 177 1.69491%	2 out of 259 0.77220%	6.23 × 10^−1^	0.000062

**Table 3:** Statistics on the FPKM values of 15 candidate genes in closed modules.

Name	C-S1	C-S2	C-S3	T-S1	T-S2	T-S3
CHS1 PB.10727.1|chr7:5288756-5290374	51.98	43.18	62.38	13.43	14.85	11.29
CHS2 PB.10728.1|chr7:5301940-5316126	3.11	4.51	3.11	15.29	7.91	12.44
CHS3 PB.10728.2|chr7:5301941-5316113	4.14	5.512	2.77	16.92	8.93	13.34
CHS4 PB.10728.3|chr7:5301944-5316192	2.16	4.15	3.10	15.34	7.13	11.24
4CL12 PB.405.4|chr1:8532291-8600510	15.32	17.61	15.33	31.62	21.21	24.34
4CL14 PB.4253.1|chr3:23869958-23873008	13.11	18.21	19.33	24.12	49.11	33.14
4CL18 PB.5838.1|chr4:349590-353192	12.12	18.16	15.13	34.17	46.21	36.15
CHR1 PB.8347.2|chr5:1481568-1493170	11.33	15.71	13.23	32.12	29.41	33.91
CHR3 PB.8348.1|chr5:1481568-1487857	13.10	18.71	13.13	35.62	29.21	26.34
CHI1 PB.2128.1|chr1:52317616-52318844	7.97	8.52	9.46	10.72	11.10	15.23
CHI2 PB.2129.1|chr1:52333401-52334681	21.12	22.81	28.53	11.22	12.90	13.01
FLS1 PB.4570.1|chr3:32763532-32765066	23.14	28.17	21.01	53.44	68.57	78.11
FLS2 PB.4570.2|chr3:32763692-32765066	14.32	19.61	16.33	44.62	39.21	36.34
F3′H PB.7377.2|chr4:42376257-42378956	5.10	7.16	3.29	16.83	14.56	13.01
F3′5′H PB.4084.1|chr3:12278062-12281867	13.01	12.59	11.74	30.45	36.18	35.16
F3′H4 PB.7478.2|chr4:42392721-42394930	12.61	11.19	17.14	39.15	33.12	31.14
FOMT PB.7235.1|chr4:37833577-37835915	53.11	43.17	51.21	15.16	19.20	17.31
ANR PB.13205.1|chr8:31320120-31332067	10.14	12.23	11.32	24.15	22.11	21.34
ANS2 PB.8440.1|chr5:3196923-3199377	16.12	16.80	14.24	35.11	36.70	34.15
ANS3 PB.8440.2|chr5:3196930-3199422	19.15	17.14	18.07	25.81	23.55	22.65
UFGT1 PB.2783.1|chr2:13439523-13441448	21.65	28.19	26.13	35.19	39.54	37.32
UFGT2 PB.2783.2|chr2:13439659-13441448	10.22	16.36	13.41	25.41	24.33	29.17
PAL5 PB.11849.17|chr7:40943081-40945873	26.13	21.75	22.98	45.21	42.76	49.81
PAL6 PB.916.1|chr1:28183235-28188005	14.23	11.10	13.43	36.29	44.11	35.18
PAL9 PB.11849.2|chr7:40942885-40959392	23.14	24.15	25.18	39.22	32.16	39.41
PAL11 PB.11849.8|chr7:40942885-40959918	24.13	22.09	23.59	50.19	54.23	55.68
DFR1 PB.339.2|chr1:7156508-7160534	22.12	23.15	26.19	40.28	44.13	45.18
DFR2 PB.340.1|chr1:7164081-7167125	20.11	21.89	22.55	39.11	36.23	39.90
UA3GT PB.6187.1|chr4:11023032-11024681	14.13	12.09	13.59	20.18	24.23	25.78
PIP2 PB.9675.6|chr5:39486362-39487950	22.11	22.19	21.49	33.29	34.22	35.24
UYP75A PB.9676.5|chr5:39463191-39464819	14.12	12.49	13.45	22.11	21.55	23.19
UYP75A PB.9848.1|chr5:43311230-43312833	13.11	11.19	13.69	21.29	24.55	22.01

## Data Availability

All data supporting the findings of this study are available from the corresponding author upon reasonable request.

## Supplementary Materials

The following supporting information can be downloaded at: https://www.mdpi.com/article/10.3390/ijms232315428/s1.
