# Supplementary material for: Comparative Transcriptome Analysis Unveils the Molecular Mechanism Underlying Sepal Colour Changes under Acidic pH Substratum in Hydrangea macrophylla
Source: Int J Mol Sci. 2022 Dec 6;23(23):15428. doi: 10.3390/ijms232315428 (PMC9739076; doi:10.3390/ijms232315428)
Supplement: Supplementary file 1 [file ijms-23-15428-s001.zip › ijms-2056783-supplementary.pdf]

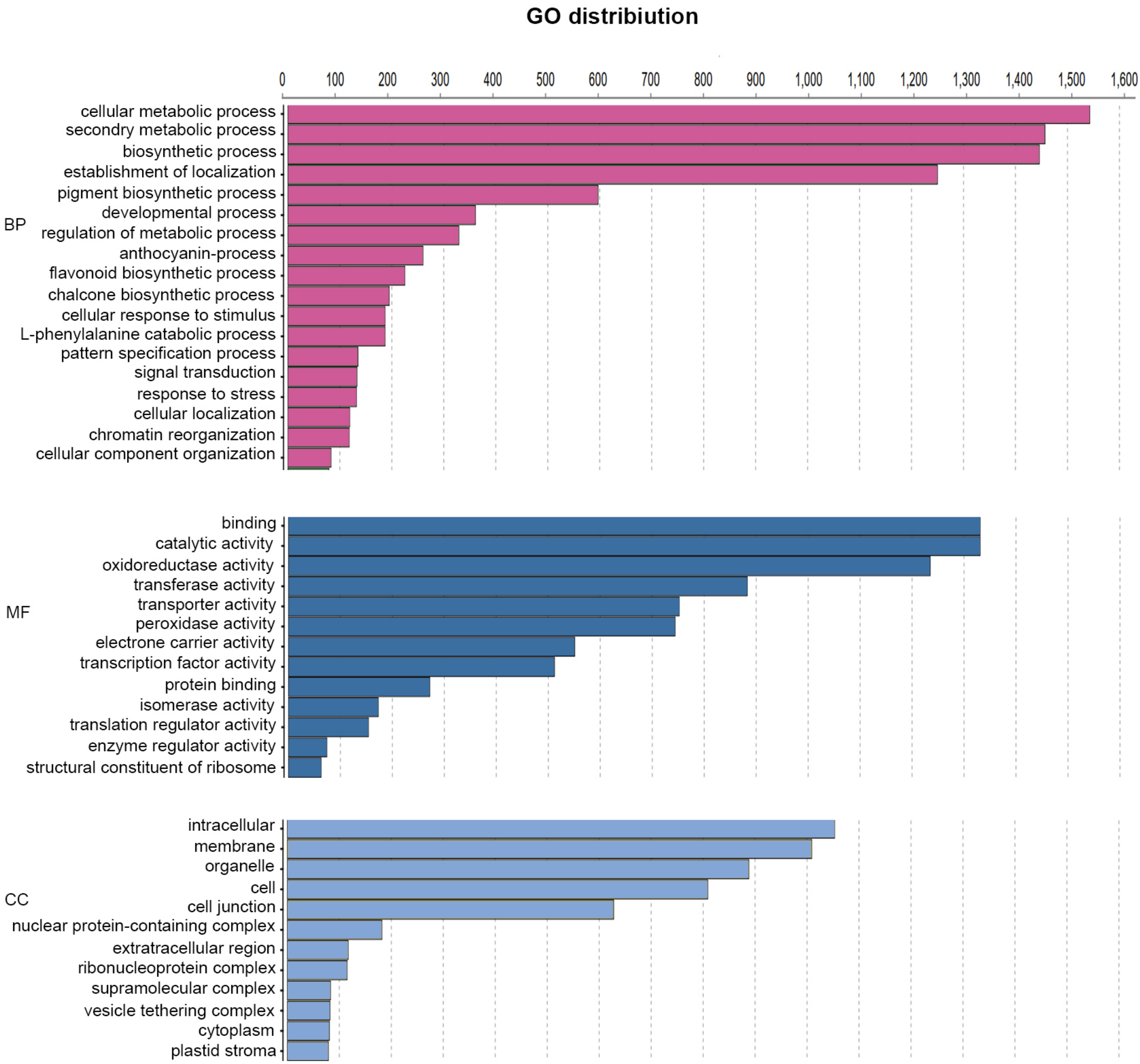

Figure S1: Functions classification of the annotated unigenes. Results are grouped by three main functional categories, Biological process, Cellular component, and Molecular function

Table S1: Primers used for RT-PCR analysis

1	Gene	F Primer sequence	R Primer sequence	Amplicon length in bp	Anneling tempreture
2	CHS1	CGCTGAAGTTGAAAGGGGATAA	TCGAAAGTGATACCAGGGCCGA	136	69.90
3	CHS2	TGCGACACTCTTCAGCTACAGAC	ACACTATGTAGGACCACGGTCTC	128	68.80
4	CHS3	ACCACGGGTGAAGGTTTGGACTG	CATCAGTTATGGAGCAATACGCA	114	58.00
5	CHI1	AAGGTGGAGAATGGGAAAGTTG	AATCGGACGAAAGCATTTGACA	128	60.00
6	CHI2	AAGGTATCTGAAATGTGCGTCG	TTGTGAAGCAAATAGAGGAGCC	137	60.00
7	F3H3	GGGTGTTCACTTTATACGAGCGGT	CCTCCCCACCTCTTCCCTGTATCT	192	60.00
8	F3H4	ACAATGGCGGAGAAGTTACGGT	GCATCATCAGATTCAACCAACA	88	60.00
9	F3′5′H	CGAAGGATTTGTGCAGGTATGAGCC	AGTTTATTTCCTTAACCCGTTCCGT	148	55.00
10	DFR1	CAAACGAGGCCAAAAATGGATTAAC	TCATTTATTACGAATTTTCGTCTCC	112	62.00
11	DFR2	AGGGCACACGTTCATCTTCTTGAGT	TCCCGTGGTGACGAGTTTTTTAGAC	100	60.00
12	UFGT	CACGGCCTCAAACTTCCTCA	ACAAGTTGCTGGTGTTGTGAAC	80	65.00
13	FLS1	TGGTGACCAAATTGAGATCCTG	TCTTTGACTTGTACTTTGGCGG	89	68.00
14	MYB12	CATTCCAAGTGGGGTAATAGAT	GTTTGCTAAGTCGTTGCTCAT	100	59.00
15	MYB2	TCATGGCACAATCTATGTATGG	TTATTTCCCCGCTTTCACTT	89	60.00
16	bHLH17	CACCACCTTCCTACCAAAACC	CCCCTTCCTTCTGCCATTC	88	60.00
17	bHLH21	CAACCGATGGAAGATAGCAC	CAACTGAGCATTTGGAGGG	103	65.00
18	WD40-1	AGCAAAAGCAGCGAGTTCTGT	AGGCGTATCGGGTTGAGGA	165	62.00
19	WD40-2	CCACATCATCCGATTTTCTCC	AACACACCTACACCACCCCAC	106	65.00
Internal control	18s	GTGACGGGTGACGGAGAATT	GACACTAATGCGCCCGGTAT	95	65.00

Table S2:Output statistics of RNA-Seq of Hydrangea macrophylla

Samples	Total Reads	Average Length (bp)	GC Percentage	Q30 Percentage
CS1-1	4496349	125	56.46%	91.76%
CS1-2	4391214	125	46.92%	92.89%
CS1-3	4412846	125	46.70%	90.94%
CS2-1	3894909	125	46.83%	91.87%
CS2-2	3783914	125	47.20%	93.91%
CS2-3	3845223	125	46.90%	90.98%
CS3-1	4559592	125	56.83%	90.87%
CS3-2	3564049	125	57.20%	90.93%
CS3-3	4659642	125	56.19%	90.55%
TS1-1	3300000	125	56.78%	90.90%
TS1-2	4071901	125	57.10%	90.78%
TS1-3	3171558	125	56.66%	90.84%
TS2-1	3893564	125	55.13%	91.87%
TS2-2	4600000	125	55.11%	91.73%
TS2-3	5272302	125	55.61%	93.15%
TS3-1	3496071	125	54.73%	91.80%
TS3-2	3000000	125	54.25%	91.88%
TS3-3	3904201	125	54.71%	92.44%

Table S3: List of TF families

MYB TF family had the most members (25), followed by WD40 (20 TFs), SPF (14 TFs), bHLH (11 TFs), C2H2 (10 TFs), ERF (5 TFs), and NAC (3 TFs) families

TFs Family	Extended name/Function
MYB0; GL1	Glabra 1
MYB11; PFG2	Production of flavonol glycosides 2
MYB111; PFG3	Production of flavonol glycosides 3
MYB113	Transcription factor MYB113
MYB12; PFG1	Production of flavonol glycosides 1
MYB2	Transcription factor MYB2
MYB23	Transcription factor MYB23
MYB27	Transcription factor MYB27
MYB4	Transcription repressor myb4
MYB5	Transcription repressor MYB5
MYB66; WER	Transcription factor MYB66
MYB7; ATY49	Transcription factor MYB7
MYB82	Transcription factor MYB82
MYBL1	MYB-like 1
MYBL2	MYB-like 2
MYBx	R3-MYB anthocyanin repressor
MYB75	Production of anthocyanin pigment 1
MYB123	Transparent testa 2
MYB90	Production of anthocyanin pigment 2
MYB114	Production of anthocyanin pigment 4
MYB32	flavone biosynthesis
MYB1	Production of anthocyanin
MYB6	Production of anthocyanin
MYBp3	flavone biosynthesis
R2R3-MYB	MYB16; MIXTA
bHLH1	bHLH1; GL3;MYC6
bHLH12	Basic helix loop helix 12
bHLH2	Basic helix loop helix 2
Bhlh4	Basic helix loop helix 4
bHLH5	anthocyanin content
bHLH001	anthocyanin content
bHLH21	anthocyanin content
bHLH17	anthocyanin content
bHLH089	anthocyanin content
bHLH062	anthocyanin content
bHLH033	anthocyanin content
WD40-1	Transcription factor WD40-1
WD40-2	Transcription factor WD40-2
WD40-3	Transcription factor WD40-3
WD40-4	Transcription factor WD40-4
WD40-5	Transcription factor WD40-5
WD40-6	Transcription factor WD40-6
WD40-7	Transcription factor WD40-7
WD40-8	Transcription factor WD40-8
WD40-9	Transcription factor WD40-9
WD40-10	Transcription factor WD40-10
WD40-11	Transcription factor WD40-11
WD40-12	Transcription factor WD40-12
WD40-13	Transcription factor WD40-13

WD40-14	Transcription factor WD40-14
WD40-15	Transcription factor WD40-15
WD40-16	Transcription factor WD40-16
WD40-17	Transcription factor WD40-17
WD40-18	Transcription factor WD40-18
WD40-19	Transcription factor WD40-19
WD40-20	Transcription factor WD40-20
NAC019	dehydration response
NAC2	regulation of transcription
NAC1	transcription factor involved in shoot apical meristem and auxin-mediated lateral root formation
AP2-ERF	positive regulator of the ABA response
AP2-ERF	Pathogenesis-related transcriptional factor
AP2-ERF	secondary cell wall metabolism
AP2-ERF	secondary cell wall metabolism
AP2-ERF	response to stress
C2H2	zinc finger protein 8 (ZFP8)
C2H2	C2H2-like zinc finger protein
C2H2	zinc finger protein9 (ZFP8)
C2H2	regulation of transcription
C2H2	Calcium-dependent ARF-type GTPase activating protein family
C2H2	Zinc finger, C2H2-type, response to photooxidative stress.
C2H2	Zinc finger, C2H2-type, salt tolerance zinc finger
C2H2	Zinc finger, C2H2-type
C2H2	Zinc finger, C2H2-type
C2H2	Zinc finger, C2H2-type
zf-HD33	regulation of transcription
zf-HD34	growth stages
zf-HD3	plant structures
zf-HD31	regulation of transcription
zf-HD21	regulation of transcription
zf-HD9	meta ion binding
zf-HD21	regulation of transcription
zf-HD22	regulation of transcription
zf-HD23	regulation of transcription
zf-HD24	regulation of transcription
zf-HD25	regulation of transcription
zf-HD26	regulation of transcription
zf-HD27	regulation of transcription
zf-HD1	regulation of transcription

Table S4: Enriched module information in TS2.

Module	Terms ID	Enriched pathways related to color formation	List hit	P-value
light green	ko00941	flavonoid biosynthesis	10	0.000657
	ko00940	phenylpropanoid biosynthesis	30	0.000012
	GO:0009813	flavonoid biosynthetic process	10	0
	ko00942	anthocyanin biosynthesis	19	0.000531
	GO:0009715	chalcone biosynthetic process	6	0.000008
brown	ko00942	anthocyanin biosynthesis	9	0.000001
	ko00943	flavonoid metabolism	2	0.000077
	ko00940	phenylpropanoid biosynthesis	7	0.000043
	GO:0045552	dihydrokaempferol 4-reductase biosynthesis	3	0.000088
	GO:0006559	L-phenylalanine catabolic process	4	0.000051

The enriched pathways related to flower color formation of each module are summarized.

Table S5: Co-expression of putative genes relatef to flavonoid biosynthesis * Significant at $p < 0.05$; ** Significant at $p < 0.01$

Gene name	#ID	Cluster-33435.149150	Cluster-33435.149151	Cluster-33435.158754	C2H2	C3H	DFR	F3'5'H	CYP73A	F3H	ANS
MYB2	Cluster-33435.66205	0.43	0.825*	0.913**	0.053	0.745*	0.853**	0.653**	-0.358	-0.183	0.652**
WDR40	Cluster-33435.149151	0.707	0.705**	0.833**	-0.072	-0.076	-0.81	-0.413	-0.525	-0.165	-0.326
WDR40	Cluster-33435.149150	0.254	0.245*	0.459**	0.289	-0.517	-0.725	-0.125	-0.315	-0.419	-0.426
WDR68	Cluster-33435.149154	0.462	0.775*	0.624**	-0.184	-0.806	-0.546	-0.541	-0.411	-0.547	-0.367
